# Taxonomy of segmental myocardial systolic dysfunction

**DOI:** 10.1093/eurheartj/ehw140

**Published:** 2016-05-04

**Authors:** Adam K. McDiarmid, Pierpaolo Pellicori, John G. Cleland, Sven Plein

**Affiliations:** 1 Multidisciplinary Cardiovascular Research Centre & Division of Biomedical Imaging, Leeds Institute of Cardiovascular and Metabolic Medicine, University of Leeds, Leeds LS2 9JT, UK; 2 Academic Cardiology Unit, University of Hull, Castle Hill Hospital, Kingston upon Hull, UK

**Keywords:** Taxonomy, Systolic dysfunction, Heart failure, Non-invasive imaging

## Abstract

The terms used to describe different states of myocardial health and disease are poorly defined. Imprecision and inconsistency in nomenclature can lead to difficulty in interpreting and applying trial outcomes to clinical practice. In particular, the terms ‘viable’ and ‘hibernating’ are commonly applied interchangeably and incorrectly to myocardium that exhibits chronic contractile dysfunction in patients with ischaemic heart disease. The range of inherent differences amongst imaging modalities used to define myocardial health and disease add further challenges to consistent definitions. The results of several large trials have led to renewed discussion about the classification of dysfunctional myocardial segments. This article aims to describe the diverse myocardial pathologies that may affect the myocardium in ischaemic heart disease and cardiomyopathy, and how they may be assessed with non-invasive imaging techniques in order to provide a taxonomy of myocardial dysfunction.

## Introduction

Studies have demonstrated the benefits of pharmacological^1–8^ and device interventions^9^ for patients with left ventricular systolic dysfunction (LVSD) of ischaemic origin. While there are clear theoretical benefits in improving blood supply to large areas of dysfunctional ‘viable’ myocardium, several recent clinical trials have failed to demonstrate improved outcomes following revascularization.^10–12^

One explanation for these results may be the terminology employed to describe myocardial health. In particular the terms ‘viable’ and ‘hibernating’ are often used interchangeably and sometimes incorrectly. ‘Viable’ is a summative term used to describe a range of myocardial states including both normal and diseased segments that nonetheless contain a substantial number of cardiac myocytes. Viable myocardial segments can thus include partial thickness scar with fairly normal cardiac myocyte function in the residual portion or myocytes that exhibit contractile dysfunction or failure. On the other hand, ‘hibernating’ myocardium refers only to the latter subgroup and can be narrowly defined as chronically dysfunctional, ischaemic myocardium that recovers contractile function following improvement in myocardial perfusion limitation. It follows that assessment of ‘viability’ can be made, to a large degree, prior to revascularization, but identification of ‘hibernation’ may only be retrospective. Furthermore, identifying myocardium as ‘viable’ does not necessarily imply ‘hibernation’ or functional recovery following revascularization, as is sometimes assumed.

A second challenge in the taxonomy of myocardial dysfunction is that it may be assessed with a range of non-invasive imaging techniques. The available techniques differ fundamentally in methodology, the properties of acquired images and the tests' limitations. Therefore, discrepancies between the modalities are inevitable, making consistency in the definition of myocardial states across imaging fields challenging. Understanding these differences is imperative, especially in the design and interpretation of studies to determine the role of revascularization in patients with long-standing LVSD.

This article creates a clinical taxonomy for the different states of myocardium that may exist and coexist in ischaemic heart disease and cardiomyopathy with reference to histology and non-invasive imaging techniques.

## Available imaging techniques

Non-invasive imaging techniques assess different aspects of myocardial health: function and morphology, perfusion, metabolism, and tissue characterization (*Table 1*).

**Table 1:** Summary of key imaging modalities, and aspects assessed in defining myocardial

	Morphology and resting function	Stress function/contractile reserve	Perfusion	Metabolism	Tissue characterization
Echo					
Strengths	Readily assessed in a range of situations. Widely accessible Doppler, tissue Doppler, and GLS provide additional important indices Contrast improves accuracy*	Physiological or pharmacological stressors may be employed			Crude visual assessment of scar, Maybe quantified with integrated background scatter (not in routine clinical practice)
Weaknesses	Limited by habitus and lung disease Greater inter-observer variation and less reproducible than CMR**	Affected by tethering in the presence of multiple wall motion abnormalities	Microbubble perfusion remains predominantly a research tool Potential lack of local expertise	Not assessed	
CMR					
Strengths	Multiplanar imaging with excellent reproducibility^#^ Myocardial mechanics may be assessed as per tissue Doppler	Physiological or pharmacological stressors may be employed Highly reproducible	Visual assessment Quantification of perfusion reserve possible Excellent spatial resolution		Focal scar identified with LGE Quantification of fibrosis (T1 mapping) and oedema (T2 mapping)
Weaknesses	Less accessible than echo Unsuitable in critical illness Limited by presence of some implantable cardiac devices & extreme obesity	Stress limitations similar to echo Exercise stressless practical Availability	Absolute quantification of perfusion Standard perfusion not ‘whole heart’ coverage Gadolinium CI if eGFR < 30 mL/min/1.73 m^2^	Not assessed in clinical routine practice, MRS available in some centres	
SPECT					
Strengths	Assessment of systolic function possible during gated perfusion examination		Whole heart coverage Perfusion defect size may be quantified		
Weakness	Other aspects of cardiac function not assessed	Not assessed	Quantitative assessment not possible Lower spatial resolution than PET and CMR Potential imitations in balanced ischaemia^‡‡‡^ Ionising radiation	Not assessed	Inferred only—scar over estimated^‡‡^
PET					
Strengths			Gold standard for perfusion quantification Excellent spatial resolution Whole heart coverage	Non-invasive assessment of carbohydrate and lipid metabolism possible	
Weaknesses	Not usually assessed	Not assessed	Exposure to ionising radiation	Exposure to ionising radiation	Tissue composition inferred from metabolism/perfusion findings^◊^

Symbols: *^136^, **^137^, #^16,17^, ‡‡^139^, ‡‡‡^140^, ◊^66^.

### Function and morphology

Echocardiography provides real-time cine assessment of cardiac structure, myocardial wall thickness, and contractile function. Tissue Doppler imaging (TDI) adds quantification of myocardial motion and global longitudinal strain can be used to detect subtle systolic and diastolic abnormalities that may have greater prognostic value compared with LVEF.^13,14^ Cardiac magnetic resonance (CMR) produces high-resolution morphological and cine images of the heart in unrestricted imaging planes and with high accuracy and reproducibility.^15,16^ In addition, cine CMR with tissue tagging or feature tracking analysis allows quantitative assessment of segmental contractile function. Both echocardiography and CMR can be combined with pharmacological (dobutamine) or physiological (treadmill/static bike) stress to detect ischaemia and viable, dysfunctional myocardium^17,18^ through demonstration of contractile reserve. Single photon emission computed tomography (SPECT) and positron emission tomography (PET) can provide information on global and regional contractile function, but at a lower resolution than echocardiography and CMR. Nuclear techniques are less suited than echocardiography and CMR to measure wall thickness. With all imaging techniques, assessment of segmental contractile reserve is significantly limited by adjacent wall motion abnormalities due to ‘tethering’.^19^

### Perfusion

**Figure 1:**
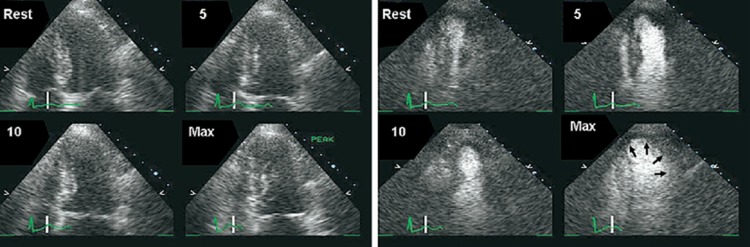
Intra-venous contrast agent use in echocardiography. Left, rest images have poorly defined endocardial border. Right, following administration of intra-venous contrast agent the apical wall motion defect is evident. Taken from Plana *et al*.^141^

**Figure 2:**
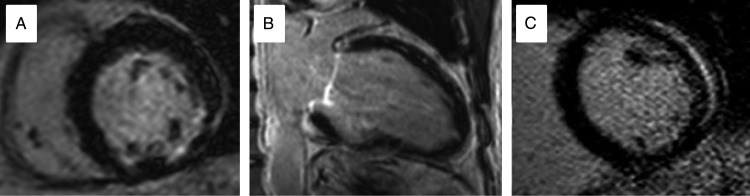
Patterns of late gadolinium enhancement by CMR seen in different pathologies. (*A*) Sub-endocardial infero-lateral enhancement in myocardial infarction. (*B*) Anterior mid wall enhancement in dilated cardiomyopathy. (*C*) Lateral epicardial and pericardial enhancement in myo-pericarditis.

**Figure 3:**
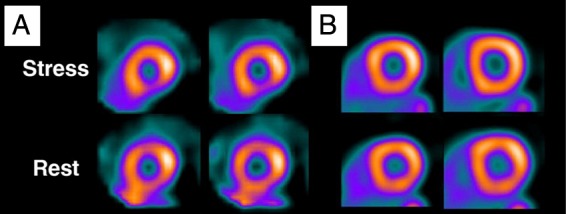
Radionuclide perfusion imaging: (*A*) Hyperaemic stress single photon emission computed tomography and (*B*) positron emission tomography imaging (*B*) of the same patient. (*A*) shows an apparent inferior perfusion defect not present in (*B*) suggesting single photon emission computed tomography attenuation artefact.^50^

Myocardial contrast echocardiography is used to measure perfusion by observing the distribution of intravascular microbubbles in the myocardium and provides a surrogate of myocardial blood flow.^20^ Myocardial perfusion CMR tracks the myocardial passage of predominantly extracellular paramagnetic contrast agents^21^ and allows estimation of absolute myocardial flow (MBF) and myocardial perfusion reserve (MPR).^22^ Single photon emission computed tomography and PET detect perfusion defects through a reduction of signal from radionucleotide bound perfusion tracers in the region of interest^23^; PET is considered the ‘gold-standard’ for quantitation of MBF and MPR (*Figures 1–3*).

### Metabolism

The principal method for metabolic myocardial imaging is currently PET, which allows myocardial metabolic substrate utilization to be characterized and quantified. Magnetic resonance spectroscopy is a less widely used method to interrogate myocardial energetics.

### Tissue characterization

Non-invasive tissue characterization is predominantly performed with CMR. Late gadolinium enhancement (LGE) CMR allows identification of scar and focal fibrosis. T1 and extracellular volume (ECV) measurement appears a useful and reproducible surrogate for diffuse myocardial fibrosis,^24,25^ while T2-weighted CMR provides assessment of myocardial oedema. Integrated back-scatter echocardiography, which provides assessment of tissue fibrosis might also be applied in this context.

### Future directions

Hybrid systems that combine anatomical with perfusion or metabolic imaging (e.g. PET/MR) are becoming available whilst molecular imaging may soon allow identification of specific biological processes.

## Taxonomy for myocardial segments

1. Normal
2. Ischaemic
  - Reversible ischaemia
        (a) Acute prolonged ischaemia
        (b) Chronic intermittent ischaemia
  - Stunning
  - Hibernation
  - Infarction
3. Myopathic

### Normal

*Definition:* Normal myocardium is by definition viable and metabolically active with normal contractile function and exhibiting contractile reserve in response to increased demand.

**Figure 4:**
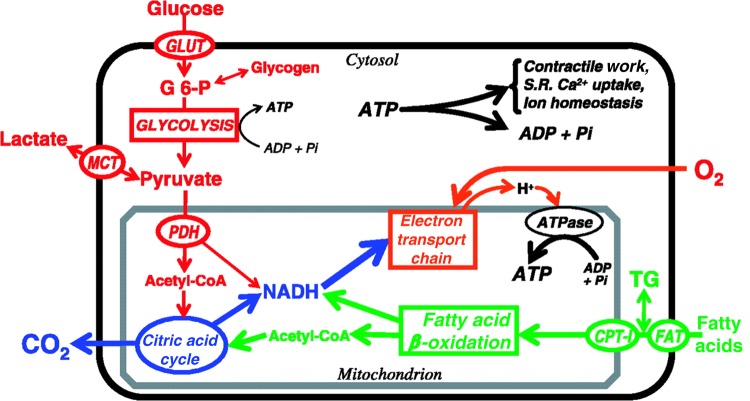
Normal myocyte metabolism: taken from Myocardial Substrate Metabolism in the Normal and Failing Heart.^28^

*Metabolism:* Normal cardiac function, including contraction, relaxation, and ionic regulation, is dependent upon adenosine triphosphate (ATP) metabolism. The majority of ATP consumption occurs in myo-fibrils throughout the cardiac cycle. Further ATP is used to regulate sarcolemmal calcium and, at the membrane, Na^+^/K^+^ ATPase transporter.^26,27^ The heart consumes ATP rapidly and is dependent upon constant renewal of ATP, which in turn is dependent upon creatine phosphate levels. Were ATP production to cease and consumption to continue unchecked, cardiac stores would be depleted in ∼10–15 s.^26^ There are three main pathways by which ATP is synthesised: fatty acid oxidation, ketone body, and carbohydrate metabolism. Fatty acid oxidation yields the most ATP, though all pathways share a common endpoint of mitochondrial ATP synthesis.^28^ In situations of increased metabolic demands, the proportion of ATP derived from carbohydrate metabolism increases (*Figure 4*).^27^

*Histology:* More than 70% of left ventricle (LV) myocardial tissue volume is cardiac myocytes; the rest is vasculature and extra cellular matrix (ECM).^29^ Predominantly containing collagen types I and III, ECM composition is regulated by a number of factors, including circulating neuro-hormones and mechanical strain.^30,31^

**Figure 5:**
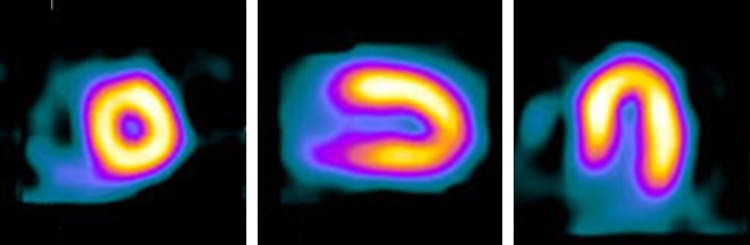
Normal single photon emission computed tomography examination: Left ventricle seen in short axis, vertical long axis, and horizontal long axis. Adapted from Hasegawa *et al.*^142^

*Imaging, morphology, and function:* The LV is a conical structure that tapers from base to apex. The normal LV wall has a thickness of between 6 and 10 mm in men, and 6 and 9 mm in women,^32^ in diastole and thickens uniformly by at least 50% in systole. In response to increasing demand, contraction becomes increasingly dynamic.

As well as function, it is also possible to assess the constituents of myocardium and tissue homogeneity. By CMR the signal in healthy myocardium is uniform on T1 and T2 weighted and contrast enhanced images. Semi-quantitative assessment of the ratio of T2 signal in healthy heart to skeletal muscle is <1.9.^33^ Quantitative measurement of the T1 signal, by T1 mapping, shows normal values in narrowly defined ranges,^34^ but depend on scanner field strength and pulse-sequence used.^34^Extracellular volume, calculated using pre- and post-contrast T1 mapping, is less method dependent and is 26 ± 3% in healthy myocardium.^24^

Nuclear techniques of perfusion and metabolism display uniform signal throughout the myocardium (*Figure 5*). Myocardial perfusion may be quantified both at rest and hyperaemia, allowing calculation of MPR. By PET normal resting MBF is ∼0.7 mL/min/g, increasing to 2.75 mL/min/g on stress, with a flow reserve of >4.^35^ ‘Gold-standard’ PET flow quantification is not without limitation, especially when the flow reduction is only mild.^36^ Estimates of absolute MBF with CMR show limited agreement with PET,^37^ though MPR correlates well with PET in health and disease,^37^ with normal MPR by CMR being ∼2.2.^38^

### Ischaemic

#### Reversible ischaemia

*Definition:* Myocardial ischaemia is a mismatch of oxygen supply and demand that precipitates change from aerobic to anaerobic cellular respiration.

*Metabolism:* Ischaemia may either be complete, due to coronary occlusion, or limited due to epicardial coronary artery stenosis or abnormalities of the myocardial microvascular circulation. The degree of ischaemia is also determined by the presence and extent of a collateral circulation that can develop in humans with established coronary artery disease.

Changes in cell metabolism begin within one minute of onset of severe ischaemia. Sub-endocardial tissue becomes ischaemic first followed by sub-epicardial tissue.^39^ Shortly after the onset of severe ischaemia, oxygen present in myocardium is consumed and normal oxidative metabolism ceases. At the same time, electron transport across cell membranes decreases and myocyte contraction becomes impaired. During this initial phase, anaerobic respiration replaces aerobic respiration as the dominant source of ATP, and glycogen replaces fatty acids and glucose as the substrate for energy production.^26^ Anaerobic respiration in this setting provides approximately one-quarter of the amount of ATP as aerobic metabolism. Due to adverse intra-cellular conditions, including falling pH, ATP production at this rate is only sustained for ∼1 min before continuing at a much lower rate for up to 1 h.^26^

In non-severe ischaemia, a degree of aerobic respiration continues, consequently more ATP is produced compared with anaerobic glycolysis. Furthermore, hydrogen ions and lactate that accumulate in severe ischaemia are produced less quickly, and ‘washed out’ of still perfused tissue, preserving a more physiological environment.

Once ischaemia has resolved, recovery of normal function is variable. Abnormalities of systolic function may persist for several days, myocardium that fails to recover normal systolic function immediately is said to be ‘stunned’.^40^

*Histology:* Abnormal function of cell membrane channels leads to myocyte oedema shortly after onset of ischaemia. In addition following short duration of severe ischaemia, depletion of glycogen stores, and the presence of ‘I-bands’ in myo-fibrils are seen on electron microscopy.^41^

*Imaging, morphology, and function*

##### Acute prolonged ischaemia

Shortly following the onset of ischaemia, regional systolic function becomes impaired and may remain so for days after the ischaemic insult.^40^ Echocardiography is most commonly used to identify wall motion abnormalities associated with acute ischaemia. Single photon emission computed tomography and PET are rarely used clinically in the setting of acute ischaemia. On CMR, wall motion and thickness are assessed in a similar fashion to echocardiography. In addition, oedema is readily detectable and quantifiable as areas of high signal on T2-weighted images.^42^ T1 and T2 mapping may provide similar, quantitative information. The oedematous zone may be quantified to determine the extent of myocardial salvage following intervention, and delineate the ‘area at risk’.

##### Chronic intermittent ischaemia

**Figure 6:**
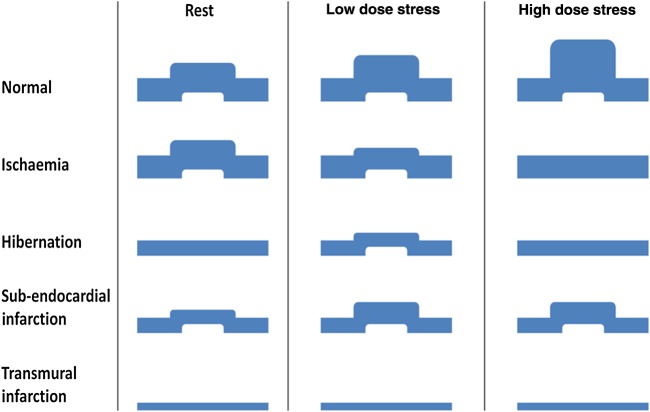
Schematic of wall motion abnormalities in ischaemia, hibernation, sub-endocardial, and infarction.

Exercise or dobutamine stress echocardiography allows detection of ischaemia as well as determining its location and extent.^43^ Ischaemic myocardium shows reduced contractile reserve with regional wall motion abnormalities developing at increasing levels of stress (*Figure 6*).

**Figure 7:**
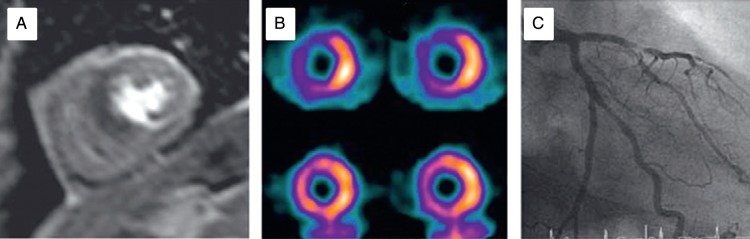
Imaging in chronic ischaemia: (*A*) cardiac magnetic resonance first pass perfusion demonstrating mid-ventricular anterior and septal abnormality. (*B*) Single photon emission computed tomography demonstrating the anterior and septal stress (top row) abnormality that resolves at rest. (*C*) Angiography in the same patient showing severe left anterior descending stenosis. Adapted from Greenwood *et al.* Cardiovascular magnetic resonance and single photon emission computed tomography for diagnosis of coronary heart disease: a prospective trial.^143^

Cardiac magnetic resonance also allows detection of ischaemia through assessment of regional systolic function. Tissue perfusion can be assessed using first pass adenosine stress CMR (*Figure 7*). Quantitative assessment of perfusion with CMR has limited agreement with PET imaging^44^ and is less commonly used in clinical practice than qualitative assessment.

Single photon emission computed tomography is commonly used in the investigation of chronic intermittent ischaemia, and while image quality on PET is superior to SPECT, availability of PET limits utility. The sensitivity and specificity of SPECT compare well with other non-invasive imaging techniques^45^; whole heart acquisition allows accurate quantification of the extent of ischaemia, a measure that may have prognostic value.^46^ Image interpretation may be limited by attenuation artefact in the inferior wall and anterior wall, especially in women.^47^ In addition to perfusion imaging and LV function, transient LV dilation (transient ischaemic dilation—TID) may be appreciated on stress SPECT imaging, marking adverse prognosis.^48^ Transient ischaemic dilation may either represent true dilation as a result of severe coronary disease and stunning, or rather may reflect sub-endocardial defects not appreciated on perfusion imaging.^49^

In head-to-head studies, the theoretical advantages of PET over SPECT have been demonstrated.^36,50^ As well as detection of ischaemia it is also possible to measure myocardial blood flow with PET,^51^ enabling quantitative assessment of myocardial blood flow reserve, which correlates strongly with coronary artery stenosis severity.^52^

#### Stunning

*Definition:* Myocardium is ‘stunned’ when contractile function is depressed following transient ischaemia, prior to a full recovery, and having sustained no irreversible myocyte damage. The mechanism of sustained systolic dysfunction in stunning is incompletely understood. However, it is believed that oxygen free radical formation and elevated myocardial calcium levels may lead to damage of myocardial proteins or sarcoplasmic reticulum.^53^

*Metabolism:* Sub-epicardial and sub-endocardial myocardial blood flow normalises quickly following restoration of normal coronary flow, however normal myocardial metabolism takes time to recover. Metabolic changes in transient ischaemia, including fall in myocyte ATP, phosphocreatine and pH, take several hours to reverse.^54^ Post-ischaemic myocardial oxidative and glucose metabolism remain depressed by ∼20% of normal levels for several hours after an ischaemic insult, and take up to 1 week to recover to near normal levels.^55^

*Histology:* Histological changes reflect sustained ischaemia. In common with metabolic changes, resolving myocardial oedema, myocardial glycogen, and ATP depletion^40^ may be detected several days later.

*Imaging, morphology, and function:* Systolic function of affected segments is impaired in stunning. The speed of recovery of systolic function is variable and may be related to the duration and severity of the ischaemic insult,^40,56,57^ Abnormalities of diastolic function, whether assessed by CMR, conventional or tissue Doppler persist beyond systolic abnormalities.^58,59^ It is likely that stunning is an under-appreciated phenomenon as functional abnormalities associated with acute ischaemia will often have recovered.

Dobutamine stress echocardiography in reperfused acute MI has been shown to predict recovery of dysfunctional segments with sensitivity and specificity of 86 and 90%, respectively.^60^

Cardiac magnetic resonance demonstrates changes in keeping with ischaemia including high signal on T2-weighted images indicative of oedema, as well as regional systolic and diastolic wall motion abnormalities.

Positron emission tomography FDG metabolism assessment may demonstrate depressed levels of glucose metabolism, but no significant metabolism perfusion mismatch, as in hibernation, is seen.^58^

#### Hibernation

*Definition:* Chronically dysfunctional viable myocardium of ischaemic origin that recovers systolic function following revascularization.

The processes underlying the development of hibernation remain unclear, although several mechanisms have been proposed. It is thought that although resting blood flow is normal, coronary flow reserves are low.^61^ This leads to repeated episodes of ischaemia and repetitive myocardial stunning, causing a complex series of physiological and structural changes characteristic of hibernation.^62^ The abnormalities seen in hibernating myocardium become more pronounced as the duration of hibernation increases.^63,64^ The time course of recovery of LV systolic function following revascularization is dependent upon the severity of myocardial change, with some studies suggesting that irreversible remodelling may occur with extended hibernation despite successful revascularization.^64,65^ However, it is not known if delayed recovery may occur beyond study duration.^66^

*Metabolism*: There remains debate regarding the metabolic changes present in hibernation. However, there is evidence to suggest that glucose uptake and utilization are increased and fatty acid metabolism is decreased in hibernating myocardium.^67,68^

*Histology:* Hibernating myocardium is macroscopically similar to normal myocardium. However, at a microscopic level, there are diffuse changes within the myocyte and extracellular ultrastructure.

**Figure 8:**
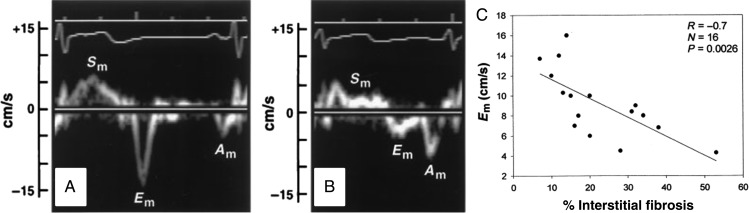
Tissue Doppler in normal *(A)* and chronically ischaemic dysfunctional myocardium *(B)*: E’ is related to the degree of interstitial fibrosis adapted from Shan *et al.*^144^

All types of collagen increase in the ECM of hibernating segments, and are more than twice that found in normal myocardium when de-differentiation is severe.^62^ Structural changes in the ECM become more pronounced as duration of hibernation increases.^64,69^ Furthermore, there is down-regulation of myocyte mitochondria and increased glycogen storage when compared with both normal and remote myocardium.^62,68^ These changes reflect alteration of mRNA expression and disorganization of cytoskeletal proteins as a result of cellular de-differentiation.

*Imaging, morphology, and function:* On functional imaging, hibernating myocardium has impaired resting systolic function, and will typically be hypo- or akinetic. Diastolic wall thickness is >6 mm by CMR^70^ or 7 mm on trans-thoracic echocardiography,^65^ though recent studies have demonstrated that thinning below these thresholds, in the absence of extensive scar, does not preclude recovery.^71^ With inotropic stimulation, hibernating myocardium shows ‘contractile reserve’ or a ‘biphasic response’, with an improvement in contractile function on low-dose/effort stress prior to deteriorating at higher workloads.^72^ Low dose stress TDI allows quantification of systolic function: Doppler tissue velocities increase more in hibernating myocardium than in dysfunctional tissue that does not show improvement in systolic function following revascularization (*Figure 8*).^73,74^

**Figure 9:**
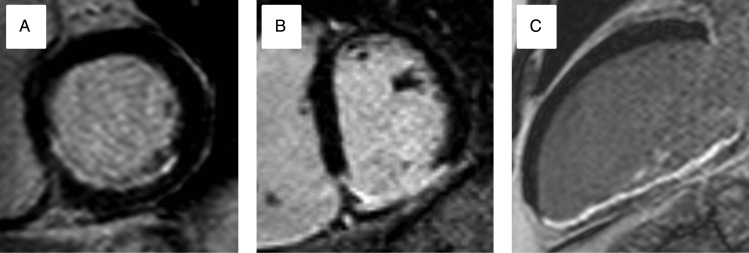
Increasing transmurality of late gadolinium enhancement predicts lack of response to revascularization in chronically ischaemic dysfunctional myocardium: Progressive transmurality of scar predicts lack of improvement in systolic function following revascularization. (*A*) 25% late gadolinium enhancement of the inferior wall and infer-septum (*B*) 50% late gadolinium enhancement in the infero-lateral wall becoming increasingly transmural inferiorly. (*C*) 100% late gadolinium enhancement of the inferior wall.

Cardiac magnetic resonance examination allows for accurate assessment of diastolic wall thickness and regional wall motion abnormalities*.* Late gadolinium enhancement CMR contributes only indirectly to the diagnosis of hibernation. On LGE CMR, hibernating myocardium has the same signal characteristics as normal myocardium, with signal uniformly nulled. Late gadolinium enhancement CMR therefore only excludes the presence of myocardial infarction as the cause of contractile dysfunction, suggesting hibernation as one of several potential causes. Absence of LGE in dysfunctional segments in ischaemic heart disease may predict functional recovery following revascularization (*Figure 9*).^75^ Cardiac magnetic resonance prediction of recovery can be further enhanced by combined use of functional, LGE, and dobutamine stress imaging. Hibernating myocardium has reduced resting function, the absence of LGE and a biphasic response to dobutamine stress.^72^ Finally, myocardial perfusion can be assessed and quantified by first pass CMR. Resting MBF (mL/min/g) is normal in hibernating myocardium, and hyperaemic blood flow is reduced with subsequent improvement following successful revascularization.^76^

In SPECT imaging cellular uptake of Thallium is dependent upon a functional Na^+^/K^+^ ATPase and preserved sarcolemmal membrane function.^66^ In hibernating myocardium, early acquisition following tracer administration identifies a defect, reflecting impaired blood flow on stress, though delayed early uptake may also be evident on rest in severe LV dysfunction.^77^ On delayed acquisitions, the isotope has been taken up by metabolically active myocytes in the hibernating region, so the defect is reduced. Stress/redistribution SPECT thus allows quantification of ischaemia and the extent of potential recovery.^78^ Technetium-based tracers, which bind within myocytes to mitochondria, identify viable myocardium similarly to Thallium SPECT.

**Figure 10:**
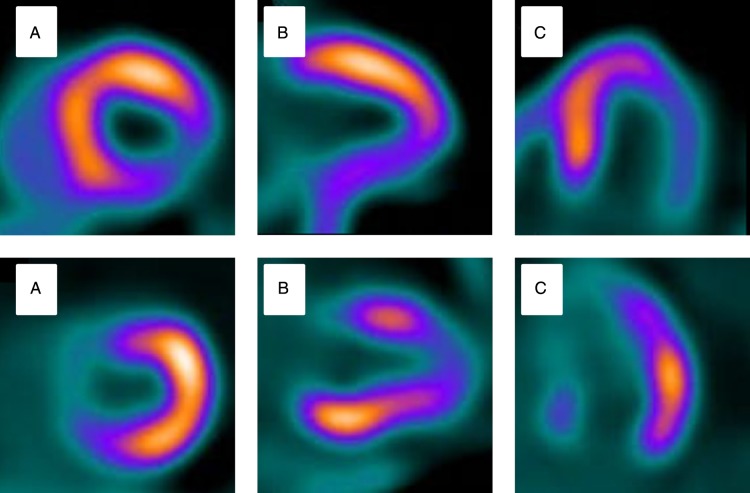
Perfusion-metabolism mismatching on PET in hibernating myocardium: (*A*) Short axis. (*B*) Vertical long axis. (*C*) Horizontal long axis. Top row demonstrates a stress perfusion defect in the infero-lateral wall. Matched metabolism imaging shows preservation of metabolism in the same territory indicating potential hibernation. Adapted from Bengel, Cardiac Positron Emission Tomography.^50^

Positron emission tomography assessment is most commonly based on the assessment of myocardial glucose uptake with FDG. Tracer signal is proportional to metabolically active myocardium and likelihood of recovery may be predicted by the glucose metabolic rate (*Figure 10*).^79,80,81^ In addition, PET allows the detection and quantification of MPR, which is reduced in hibernation.^82,83^

#### Hibernation with non-trans-mural scar

Hibernation commonly co-exists in the presence of sub-endocardial infarction and as a consequence overlap of imaging findings may be seen, e.g. sub-endocardial enhancement on LGE CMR, or both fixed and inducible perfusion defects on nuclear study.

In the setting of partial infarction response to dobutamine stress on functional imaging is reduced. Decreased circumferential strain measured by echocardiography further facilitates differentiation of normal myocardium, sub-endocardial, and transmural infarction.^84,85^

The spatial resolution of CMR LGE enables the detection of small volumes of infarction that may be missed by SPECT or PET. Transmural extent of LGE predicts likelihood of systolic recovery: 60% with 1–25% LGE, 42% with 26–50% LGE, and only 7% of segments with >50% enhancement recovered at 3 months in a cohort with predominantly chronic, mild LVSD (LVEF 43%).^75^ In a second mixed cohort of acute and chronic patients with moderate LVSD (LVEF 38%) recovery rates were similar (0 LGE:73%, 1–25:56%, 26–50:45%, >50%:5%).^86^

Where hibernation co-exists with acute abnormalities a ‘mixed picture’ of imaging findings may result. Definitive classification of abnormalities in this setting is difficult and may require repeated imaging to classify myocardium.

#### Infarction

*Definition:* Myocardial infarction follows sustained ischaemia leading to myocyte necrosis and subsequent remodelling and fibrosis.

*Metabolism:* Necrosis occurs when sustained severe ischaemia leads to irreversible structural changes within the myocyte, including mitochondrial swelling and disruption of the sacrolemma.^41^ In the course of ischaemic injury, necrosis begins in the sub-endocardium where tissue perfusion is lowest and energy consumption is highest, leading to ATP supply exhaustion and accumulation of the by-products of glycolysis. Sub-endocardial infarction commences ∼20 min after ischaemia onset. Prolonged ischaemia leads to increasingly transmural necrosis, which moves as a ‘wave front’ from the endocardium to the epicardium,^39^ though sometimes with sparing of a thin rim of sub-endocardium.

*Histology:* Characteristic histological changes occur during myocardial infarction, and evolve until the infarcted region undergoes scar replacement. On macroscopic examination there are few detectable changes over the first 4 h. From 4 to 12 h myocardium becomes mottled. Over the next week, the infarct centre becomes pale and yellows while developing red margins, followed by replacement of necrotic infarct by fibrous scar tissue.^87,88^

**Figure 11:**
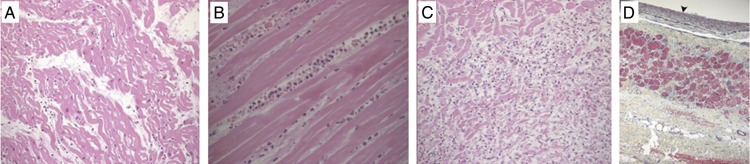
Histological change following myocardial infarction: pathology of myocardial infarction, diagnostic histopathology 2013. (*A*) Light microscopy of wavy mitochondrial fibres and interstitial oedema 4 h postmyocardial infarction. (*B*) Myocardial fibres 24 h post-myocardial infarction, myocardial thinning, and interstitial infiltration by polymorphonuclear leukocytes. (*C*) Granulation tissue 7–10 days post infarction. Near complete removal of myocytes. (*D*) Light microscopy 2–3 weeks after infarction. Subendocardial fibrosis marked by arrowhead, with a small area of myocardial fibre preservation between layers of collagen deposition. Adapted from Chang *et al*.^145^

Microscopically, myocardium undergoes a series of changes responsible for the macroscopically appreciable abnormalities. During the initial phase, glycogen depletion and oedema, in keeping with severe ischaemia is seen on electron microscopy.^89^ Between 4 and 12 h, oedema, necrosis, and intra-myocardial haemorrhage are seen. From 12 to 24 h neutrophil infiltration and ongoing necrosis develop. This is followed within 24–48 h by the disappearance of nuclei and striations, and macrophages remove dead cells at the infarct border. Following this granulation tissue and collagen deposition begins, eventually leading to the formation of collagen scar (*Figure 11*).^90,91^

*Imaging, morphology, and function:* Wall motion abnormalities develop before ECG changes or symptoms,^92^ while TDI systolic velocities decrease rapidly following onset of ischaemia.^93^ Following transmural infarction, myocardium is akinetic and thins with time. On stress or exercise there is no improvement in wall thickening with the absence of contractile reserve.^94^ Sub-endocardial infarction results in wall motion abnormalities of varying severities, and may be differentiated from transmural infarction using peak systolic circumferential strain and strain rate on stress echocardiography.^95^

**Figure 12:**
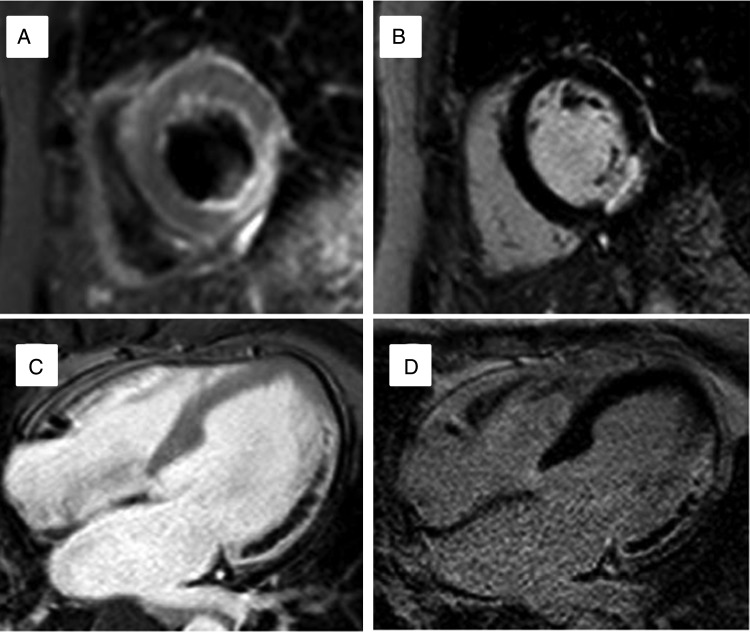
Acute myocardial infarction on cardiac magnetic resonance: Top row: (*A*) High signal on T2-weighted image demonstrating Infero-lateral oedema and ‘area at risk’. (*B*) Subendocardial infarction in the same patient on late gadolinium enhancement. Bottom row: (*C*) early gadolinium enhancement image in a second patient with extensive lateral wall hypoenhancement. (*D*) Late gadolinium enhancement confirms infarction and microvascular obstruction.

Cardiac magnetic resonance demonstrates gross anatomical changes associated with infarction including remodelling, wall thinning, and motion abnormalities.^42^ In addition, CMR imaging can be used to delineate infarct extent, indicate the area at risk, and detect microvascular and reperfusion injury.^96,97^ Late gadolinium enhancement CMR after acute MI displays high signal in the infarcted area due to persistence of gadolinium within areas of increased extracellular volume and abnormal washout kinetics.^42^ In animal models, infarct enhancement with LGE CMR has been observed within 90 min of the onset of ischaemia.^98^ In the acute phase, infarct extent may be overestimated due to increased signal and contrast in the oedematous peri-infarct zone.^99^ In acute myocardial infarction, LGE CMR may show an area of low signal within the otherwise high-signal infarct zone. This reflects microvascular obstruction (MO) leading to an absence of gadolinium delivery to the centre of the infarct. Extensive MO is associated with no-flow on invasive coronary angiography and adverse LV remodelling.^100^ Within an area of MO, myocardial haemorrhage may occur, causing low signal on T2 and T2* CMR.^101^ Haemorrhage represents more severe structural change in the setting of acute MI and is also associated with adverse LV remodelling following infarction.^102^ The combination of the information gained from T2 weighted, early gadolinium, and late gadolinium images allows measurement of the area at risk, infarct size, and myocardial salvage (difference between area at risk and infarct size) (*Figure 12*).^97^

At the time of acute infarction SPECT imaging is not usually performed. However in research settings, infarct area as delineated by absent tracer uptake on SPECT correlates well with infarct size on pathological specimens.^103^ In chronic infarction, metabolically active myocytes are replaced by scar and the absence of an intact Na^+^/K^+^ ATPase leads to lack of uptake of the SPECT tracer within the region of infarction. The limited spatial resolution of SPECT can lead to an under-appreciation of the extent of infarction because small sub-endocardial infarction may not be detected.^104^

In acute myocardial infarction, FDG PET allows detection of infarction and the presence of viable tissue in or adjacent to the infarcted territory. The absence of detectable glucose metabolism is associated with irreversible myocardial injury.^105^ Myocardial perfusion by PET in the acute MI zone is depressed, significantly improves following coronary intervention, and may continue to improve up to 2 weeks later.^106^

In the setting of chronic myocardial infarction, concordant reduction in signal from both perfusion and metabolism (NH3 and FDG tracers, respectively) PET is seen and readily appreciated in trans-mural infarction; however, in sub-endocardial infarction PET may fail to detect small areas of sub-endocardial scar when compared with CMR.^107^

### Myopathic myocardium

*Definition:* Dysfunctional myocardium of non-ischaemic origin is considered to be myopathic. Myopathy covers a wide range of pathologies. This review will focus on dilated cardiomyopathy, in which pronounced isolated segmental systolic dysfunction is uncommon, with only passing reference to other aetiologies.

*Metabolism*: Normal cardiac function relies upon matching of energy demand and consumption. This requires an appropriate oxygen supply to myocytes, mitochondrial function, ATP transport to the site of energy consumption, and a reliable feedback system to maintain appropriate metabolic rates. Energy depletion, perhaps due to mitochondrial dysfunction, could be a primary reason for impaired myocardial contraction. However, the primary defect may usually be in the contractile apparatus or in calcium handling, which increase metabolic demand. Importantly, myopathy may be an acquired phenomenon in un-infarcted myocardium that has undergone remodelling and may co-exist alongside segments affected by scar, ischaemia, stunning or hibernation. In patients with mild heart failure secondary to idiopathic dilated cardiomyopathy any change myocardial substrate utilization is subtle. In severe heart failure, cellular metabolism changes substantially, with greater glucose and less free fatty acid utilization,^108,109^ in most but not all studies.^110^

**Figure 13:**
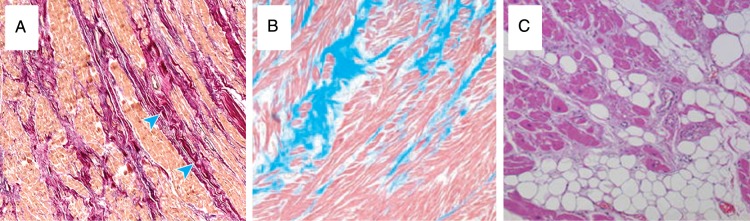
Histological abnormalities in cardiomyopathy: (*A*) Dilated cardiomyopathy, increased interstitial fibrosis at the blue arrow. (*B*) Hypertrophic cardiomyopathy, increased interstitial fibrosis (blue), and myocyte disarray. (*C*) Fibro-fatty replacement in arrhythmogenic right ventricular cardiomyopathy. Taken from Hughes, Asimaki and Saffitz, Gulati.^146–148^

*Histology:* Histological abnormalities differ depending on the underlying aetiology including myocyte disarray and interstitial fibrosis^111^ in hypertrophic cardiomyopathy (HCM) and fibro-fatty replacement in arrhythmogenic right ventricular cardiomyopathy. A reduction of mitochondria in the failing heart is common.^28^ Differing degrees of myocyte hypertrophy are seen depending upon the aetiology of heart failure. However irrespective of aetiology, a common finding is the expansion of the ECM and fibrosis due to local factors and activation^112^ of the renin–aldosterone–angiotensin system.^113^ Collagen types I and III are the major structural components in the cardiac ECM, providing both tensile strength and elasticity.^114^ In the failing heart, collagen synthesis increases (especially type III collagen), leading to accumulation of intercellular collagen, limiting ventricular compliance, myocyte function, and contributing to both systolic and diastolic dysfunction (*Figure 13*).^115,116^

*Imaging, morphology, and function:* Functional imaging by echocardiography and CMR supplies important information for prognosis and risk stratification including ejection fraction and left ventricular end diastolic dimensions.^117,118^

Comprehensive structural assessment by echocardiography and CMR may guide management and allow diagnosis of the underlying cardiomyopathic process. For example, it is possible to differentiate between morphologically similar cardiomyopathies on echocardiography: Improvement in long-axis function on stress TDI allows differentiation between ischaemic and non-ischaemic cardiomyopathy.^119^ In the case of left ventricular hypertrophy, echocardiographic 2D-strain assessment allows differentiation of HCM and hypertensive LV hypertrophy, while TDI enables differentiation of HCM and athlete's heart.^120,121^

Tissue Doppler imaging of the mitral annulus and mitral inflow velocity provides a non-invasive estimate of left atrial pressure.^122^ Cardiac magnetic resonance assessment of left atrial transit time also correlates strongly with LV early diastolic pressure.^123^

In many cardiomyopathies, LGE CMR shows characteristics patterns of enhancement. The presence and extent of LGE predicts outcomes in a range of cardiac diseases, including DCM,^124^ HCM,^125^ and ischaemic heart disease.^126^ Extracellular volume calculation allows measurement of both diffuse fibrotic and infiltrative processes that are unreliably assessed with visual analysis alone.^127,128^ T2* mapping allows detection and tracking of iron overload cardiomyopathy.^129^

The ability of SPECT alone to differentiate ischaemic from DCM is uncertain as mild stress perfusion defects are commonly seen with both aetiologies.^130,131^ The defects seen have mild stress defect severity ratios (>45%); however, similar abnormalities may be seen in multi-vessel coronary disease precluding the use of SPECT as the sole diagnostic tool in this situation.

Decreased free fatty acid metabolism with increased glucose metabolism is found on PET examination in DCM.^108^ Hyperaemic blood flow by PET is lower in DCM than in healthy controls, 2.23 mL/min/mL vs. 4.33 mL/min/mL in one report^132^ and perfusion abnormalities in DCM are progressively worse in more severe heart failure^133^ and carry adverse prognosis.^134^

## Clinical implications

Different imaging modalities assess different facets of myocardial health and disease and are often complementary. An awareness of the principles underlying acquisition, and the aspect of myocardial health and pathology assessed is crucial.

The detection of ‘normal’ myocardium is straightforward, and can be accomplished using any test capable of delivering good quality anatomical images. The most appropriate method will vary depending upon patient and institute: In terms of practicality, availability, and economy this will frequently be echocardiography. Atrial as well as ventricular volumes and function should be carefully assessed to detect more subtle disease. Minor deviations from normal, such as small areas of infarction, may be undetected unless high-resolution imaging methods such as LGE CMR are used.

Ischaemia detection or perfusion assessment may be performed using either stress/exercise echo/CMR, CMR first pass perfusion or nuclear imaging, and all are included in current practice guidelines.^135^ Single photon emission computed tomography is the mostly widely used modality worldwide, and PET remains the gold standard. In some patients, it may be desirable to exclude valvular disease or cardiomyopathy making DSE or CMR the most appropriate choice of test.

Hibernation may only ever be identified retrospectively. However, in clinical practice the question most often posed relates to the likelihood of contractile recovery in a given coronary territory, or the potential for LV reverse remodelling following revascularization. For this purpose, any of the non-invasive imaging techniques covered may be selected, as long as the limitations of the chosen technique are recognized in decision making. However, randomized controlled trials have generally not shown a greater improvement in either ventricular function or prognosis with coronary revascularization compared to pharmacological treatment in patients with heart failure and a reduced LV ejection fraction who have greater myocardial viability,^10,11,149^ although extended follow of the STICH population recently showed modest 16% reduction in mortality after a median follow-up of 10 years (*p* = 0.019).^150^ This may reflect a failure of previous taxonomies rather than a failure of concept; further randomized controlled trials are required.

Partial infarction is best appreciated by CMR examination, with the added benefit that systolic abnormalities not associated with coronary disease may be explained by characteristic abnormalities of cardiomyopathy on LGE CMR.

Where infarction and cardiomyopathy co-exist, multi-modality imaging may be necessary, often including coronary imaging to better understand both the dominant aetiology of LVSD and potential for recovery. Tissue Doppler imaging velocities have been shown to differ in DCM and ischaemic heart disease, potentially allowing discrimination of causes of LVSD. Late gadolinium enhancement CMR allows the extent of scarring due to either pathology to be determined, but not the benefit of any specific therapy.

Mild perfusion defects, with stress defect severity ratios of >45%, are common in DCM as discussed above. SPECT, PET, or CMR first pass perfusion in combination with coronary angiography may facilitate targeted revascularization, if indicated on conventional grounds, and avoiding unnecessary revascularization.

## Conclusion

**Figure 14:**
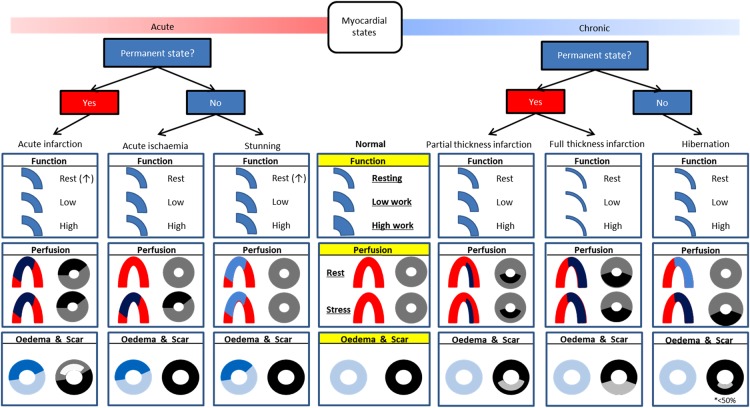
Taxonomy of myocardial segments in left ventricular systolic dysfunction: This should be viewed as an aid to classification rather than a decision tree. Function: thickness compared with ‘normal’ denotes resting state, with subsequent contractile reserve displayed with increase in segmental thickness. Stunned myocardium may display increased thickness at rest due to oedema though may not be readily appreciable. Perfusion: ‘horseshoe’ displays typical Single photon emission computed tomography/positron emission tomography finding whilst ‘donut’ displays cardiac magnetic resonance findings: Single photon emission computed tomography/positron emission tomography: Red = normal; pale blue = minimally decreased or normal; dark blue = decreased. Cardiac magnetic resonance: grey = normal; black=hypoperfusion/ischaemia. Oedema & Scar: ‘donut’ displays typical T2 weighted (blue) and late gadolinium enhancement findings (black is normal, shades of grey represent late enhancement).

Consistent adoption of standard nomenclature in clinical practice will facilitate thinking and hopefully decision making regardless of local access to different imaging modalities. *Figure 14* summarizes key imaging findings covered and aims to provide a reference for future studies. A clear taxonomy for myocardial viability and dysfunction provides the basis for randomized controlled trials that will provide the scientific evidence upon which to base clinical decisions.

### Funding

A.K.M. is funded by a British Heart Foundation (BHF) Project Grant (PG/14/10/30641). S.P. is funded by a BHF Clinical Fellowship (FS/10/62/28409). Funding to pay the Open Access publication charges for this article was provided by The British Heart Foundation (BHF).

**Conflict of interest:** none declared.
